# Immunohistochemistry Analysis in Inflammatory Bowel Disease—Should We Bring to Light Interleukin-10?

**DOI:** 10.3390/biomedicines13020406

**Published:** 2025-02-07

**Authors:** Christopher Pavel, Mircea Mihai Diculescu, Madalina Ilie, Oana-Mihaela Plotogea, Vasile Sandru, Valentin Enache, Dan-Ionut Gheonea, Alexandra Jichitu, Alexandru Constantinescu, Robert-Emmanuel Serban, Cosmin Viorel Bogu, Horia-Dan Liscu, Alex-Emilian Stepan

**Affiliations:** 1 Department 5, Gastroenterology, Carol Davila University of Medicine and Pharmacy, 050474 Bucharest, Romania; christopher.pavel@gmail.com (C.P.); drmadalina@gmail.com (M.I.); plotogea.oana@gmail.com (O.-M.P.); drsandruvasile@gmail.com (V.S.); alexandruconstantinescu1991@gmail.com (A.C.); 2 Department of Gastroenterology, Clinical Emergency Hospital of Bucharest, 014461 Bucharest, Romania; jichitualexandra@yahoo.com (A.J.); cosmin-viorel.bogu@rez.umfcd.ro (C.V.B.); 3 Department of Gastroenterology, Fundeni Clinical Institute, 022328 Bucharest, Romania; 4 Department of Pathology, Clinical Emergency Hospital of Bucharest, 014461 Bucharest, Romania; valienache@rocketmail.com; 5 Department of Gastroenterology, University of Medicine and Pharmacy of Craiova, 200349 Craiova, Romania; digheonea@gmail.com (D.-I.G.); drrobert.serban03@gmail.com (R.-E.S.); 6 Discipline of Oncological Radiotherapy and Medical Imaging, Carol Davila University of Medicine and Pharmacy, 050474 Bucharest, Romania; horia-dan.liscu@drd.umfcd.ro; 7 Department of Pathology, University of Medicine and Pharmacy of Craiova, 200349 Craiova, Romania; astepan76@yahoo.com

**Keywords:** inflammatory bowel disease, immunohistochemical parameters, biologic treatment

## Abstract

**Background/Objectives:** Inflammatory bowel diseases (IBDs) are chronic intestinal disorders with an unpredictable course. In parallel with the advent of new biologic therapies targeting specific interleukin pathways, end-point targets have become more stringent, aiming for mucosal and even histologic healing. **Methods:** We conducted a prospective study assessing immunohistochemical (IHC) parameters in 46 IBD patients treated with biologic therapy. A similar IHC analysis was performed for comparison with a cohort of 10 “non-IBD” patients. **Results:** The highest integrated optical density (IOD) of TNF-α was observed in patients with dysplasia, abscesses, mucin depletion and basal plasmacytosis. Non-responders had higher pre- and post-treatment TNF-α expression in both UC and CD compared to responders. On the contrary, the same analysis conducted in the subpopulation treated with anti-TNF-α therapy (Infliximab and Adalimumab) did not reveal a substantial difference in TNF-α expression between responders and non-responders. High pre-treatment interleukin-10 expression was associated with biologic therapy failure, histological inflammatory activity and longer disease duration. **Conclusions:** Pre-treatment assessment of IL-10 might be a useful tool for identifying a high-risk subset of IBD patients and determining a more aggressive therapy and intensive monitoring strategy.

## 1. Introduction

Inflammatory bowel diseases (IBDs) are chronic intestinal disorders with an unpredictable course despite rigorous scientific struggle targeting new molecules and treatment strategies [1,2]. The introduction of anti-tumor necrosis factor (TNF) therapy represented a quantum leap, but the highly intricate pathophysiology is still responsible for a 30% primary loss and up to a 50% secondary-loss of response [3,4,5]. As a consequence, recent data refute significant declines in hospitalizations or surgical intervention rates [6]. In parallel with the advent of new biologic therapies targeting specific interleukin pathways, end-point targets have become more stringent, aiming for mucosal and even microscopic (histologic) healing [7,8,9,10]. The latter has a clear benefit in terms of lower rates of relapse, corticosteroids use, hospitalizations and colorectal cancer [11,12,13].

Alongside environmental exposures, gut microbiota and genetic alterations, immunological mechanisms have a significant impact in the pathophysiology of IBD [14,15], leading to an imbalanced release of pro- and anti-inflammatory molecules [16]. This cytokine-cell network plays an essential role in cell signaling and the initiation and perpetuation of intestinal inflammation [17]. Apart from the well-established anti-TNF therapy, novel molecules target specific pathways involved in the chronic inflammatory response (i.e., anti-interleukin (IL)-12/23, anti-α_4_β_7_ integrin antibody or JAK inhibitor molecules).

The scientific background behind the expression of cytokines in inflamed mucosa has evolved over the last decades. Interleukins are a type of cytokine produced by macrophages, T lymphocytes, neutrophils, mast cells and epithelial cells [17,18]. Cytokines, such as TNF-α, IL-1β and IL-6, can drive tissue damage [19,20,21], whereas IL-10 is typically recognized as an anti-inflammatory molecule [18,22,23]. A plethora of research has elucidated the convoluted network of pro-inflammatory cytokines (TNF-α, IL-1β and IL-6), along with downstream effectors and signal transduction mechanisms, leading to inflammation, apoptotic cell death, intestinal permeability, histological damage, fibrosis and carcinogenesis [24,25,26,27,28].

The success of anti-TNF treatment in IBD is undisputable, but the rate of non-response is persistently high despite different monitoring strategies [29]. With the ongoing expansion of the therapeutic armamentarium, identifying IBD patients who will benefit from anti-TNF therapy and evaluating therapeutic response remain major targets in IBD management [30,31,32]. TNF-α expression in ileo–colonic macrophages was discovered in both CD and UC [33] and plays a fundamental role in the induction and maintenance of intestinal inflammation [30,34]. The IL-1 family (IL-1α and IL-1β) has immunological up-regulatory and pro-inflammatory activity [35]. It was previously shown that colonic macrophages in IBD patients activate ICE (IL-1 converting enzyme), releasing mature IL-1β into colonic mucosa [36].

IL-6 contributes to the differentiation of T helper 17 (Th17), which is involved in the pathogenesis of IBD [37,38,39]. In acute inflammation, endothelial cells, monocytes and macrophages release IL-6, which triggers the expression of adhesion molecules and chemokines involved in neutrophil recruitment [37,40]. In chronic inflammation, IL-6 increases the survival of neutrophils by reducing apoptosis [37,41].

IL-10 is an immunomodulatory cytokine that inhibits antigen presentation and the release of pro-inflammatory cytokines [35]. Inactivation of IL-10 in mice leads to increased IL-12 and IFN-γ, whereas inflamed tissues exhibit low IL-10 [42]. According to Neurath et al., loss-of-function mutations in the genes encoding IL-10 and the IL-10 receptor are associated with very early-onset IBD, whereas a lack of IL-10 triggers episodes of spontaneous colitis [20].

The aim of this study was to prospectively assess the immunohistochemical parameters of the main inflammatory cytokines involved in the pathophysiology of IBD. Vigorous research has not previously been conducted in prospective trials on the molecular expression of these cytokines in different phases of disease (before and during treatment) to assess the impact of medical therapy. Simultaneously, there is a remarkable paucity regarding immunohistochemical and histological correlations in biopsy samples from IBD patients. The current research addresses these important gaps in the literature while also aiming to identify a subset of high-risk patients.

## 2. Materials and Methods

### 2.1. Study Design

This was a prospective study assessing the immunohistochemical (IHC) parameters of 46 IBD patients treated with biologic therapy at Bucharest Clinical Emergency Hospital at two stages of their disease course. A cohort of 10 non-IBD patients (with a normal colonoscopy report) underwent a similar IHC analysis to increase statistical power.

Immunostaining focused on:

- Inflammatory cytokines TNF-α, IL-1β, IL-6 and IL-10. Apart from IL-6, which had merely cytoplasmic reactions, TNF-α, IL-1β and IL-10 had predominantly cytoplasmic and some focal, incomplete membranous reactions.
- The p53 positivity index and Ki-67 proliferation index. The positivity of Ki-67 and p53 was only reported for the epithelial component.
- In the first phase, biopsies were collected before the initiation of biologic therapy, either de novo or after switching to another class due to previous primary/secondary loss of response. Biologic therapy is an umbrella-term used in our study for both biologic and small-molecule drugs.
- In the second phase, patients were reassessed after 4–6 months to compare IHC and histological findings.

For a thorough evaluation, correlations between histology and immunohistochemical staining patterns were also analyzed. Histopathological findings were assessed using the Nancy Index. The following parameters were examined: crypt architectural distortion, basal plasmacytosis, Paneth/pyloric cell metaplasia, lymphoid aggregates, cryptitis, crypt abscesses, ulcerations and mucin depletion (evaluated as present or absent).

We also assessed fecal calprotectin as a biological marker of inflammation.

The enrollment period was between 1 March 2023 and 29 February 2024, and the last evaluation was performed in August 2024.

- Inclusion criteria (IBD cohort):
  ○ Patients were considered for enrollment when their disease course required the initiation of biologic therapy, either as an escalation from conventional therapy (biologic-naive patients) or as a switch to another class due to primary/secondary loss of response (biologic-experienced patients). Therapy was guided in accordance with the current recommendations published by the European Crohn’s and Colitis Organization (ECCO) [43,44].
- Inclusion criteria (non-IBD cohort):
  ○ Patients with normal findings after colonoscopy, with no signs of inflammatory or tumoral pathology.
  ○ In order to increase accuracy, the mean age of the non-IBD cohort was similar to that of the IBD cohort.
- Exclusion criteria:
  ○ Lack of informed consent or a lack of will to comply with the scheduled follow-up.
  ○ Histopathological diagnosis of microscopic colitis (for patients in the non-IBD cohort with a normal colonoscopy report).
  ○ Insufficient tissue samples for adequate immunostaining analysis.

Immunohistochemical analysis was performed by one expert GI pathologist from the Pathology Department of University of Medicine and Pharmacy, Craiova. The antibodies, clones, antigen retrievals and external positive controls are summarized in Table 1.

In order to improve the accuracy of immunohistochemistry evaluation, a semi-quantitative integrated optical density (IOD) analysis was performed for IL-1β, IL-6, TNF-α and IL-10, since manual interpretation of IHC and the reproducibility of scoring systems are highly subjective [45]. IOD was computed as pixel area × mean intensity. The slides were scanned at high power magnification (40×) using the Motic EasyScan Pro 6 (Motic Digital Pathology, Barcelona, Spain). To assess the immunostaining signal, images from each slide were exported as JPEG files (Motic DSAssistant). The IHC signal was quantified as an area or number of positive cells using Image-Pro Plus 7 (Media Cybernetics, Bethesda, MD, USA). For each slide, the signal expression area and IOD were recorded from three different images, and the final value was reported as the mean IOD.

The Ki-67 proliferation index and p53 positivity index were computed as positive epithelial cells/total epithelial cells (magnification 40×). In each case, positive cells from five different microscopic fields were counted from the ‘hot-spot’ staining areas. The final mean value was used for the analysis.

This study was approved by the local Ethics Committee (registration number 45027) on 12 October 2022, in accordance with the Health Minister Order, 1502/2016. All patients provided written informed consent.

### 2.2. Statistical Analysis

Statistical analysis was performed using the SPSS 20.0 v.20 (IBM, Armonk, NY, USA) software package. A *p*-value below 0.05 was considered to be statistically significant. The continuous variables were expressed as the means ± standard deviations and the ranges as medians and min–max ranges. To assess differences between the two groups, *t*-tests were computed using the scipy.stats library [46] in Python (version 3.7). Paired *t*-tests were used to compare data from the same patients before and after treatment, otherwise unpaired *t*-test were used. To compare multiple groups, a one-way ANOVA with Tukey HSD Post-Hoc Analysis was employed using the statsmodels library [47]. The distribution of categorical variables was assessed using the chi-square test included in scipy.stats library. Correlations were calculated using the Pearson correlation method implemented in scipy.stats. The correlation results for parameter pairs were assembled into a matrix, and hierarchical clustering of the parameters was performed using seaborn [48].

## 3. Results

### 3.1. Study Population

**Figure 1:**
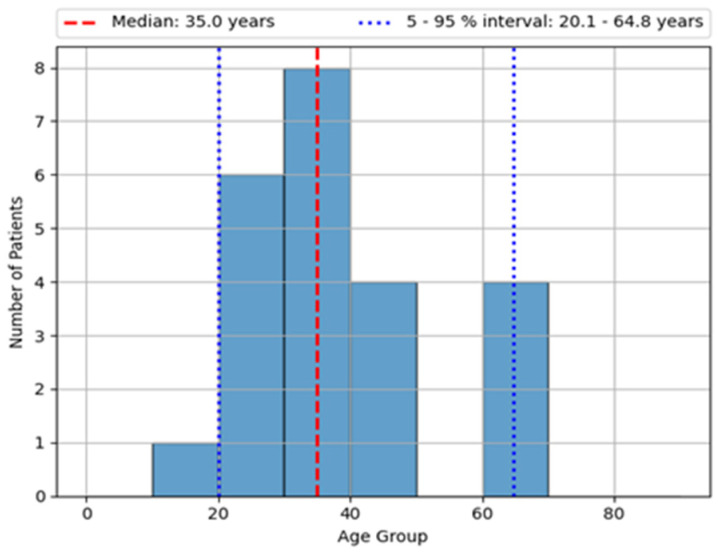
Mean age of IBD patients.

The mean age of IBD patients was 35 years old (Figure 1), whereas the mean age at the time of IBD diagnosis was 32 years old. The male-to-female ratio was relatively similar, with a slight overall male preponderance (52.2%). Approximately one-third of patients (30.4%) were biologic-experienced at the time of study enrollment (Table 2).

In terms of phenotype according to the Montreal classification [49] (Table 3), CD patients were most frequently classified as having colonic disease (L2, 38.5%), followed by ileocolonic (L3) and ileal disease (L1), which had equal distribution (30.8%). The most common behavior was stricturing (B2, 69.3%), followed by penetrating (B3) and inflammatory patterns (B1), with similar percentages (15.4%). In UC, pancolitis (E3) was diagnosed in 50% cases, followed by extensive colitis (E2) in 40% cases, whereas proctitis (E1) was observed in the remaining 10% of cases.

### 3.2. Immunohistochemical Analysis

#### 3.2.1. TNF-α

TNF-α expression in UC patients was significantly higher before treatment compared to the control group (with a *p*-value of 0.02). A similar statistical significance was not reached in CD patients, where TNF-α expression was lower compared to UC patients.

**Figure 2:**
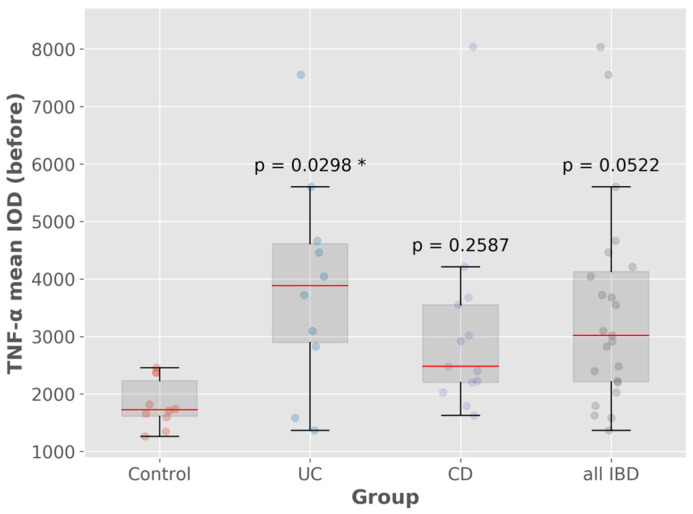
TNF-α mean IOD (control vs. IBD). * *p* < 0.05.

**Figure 3:**
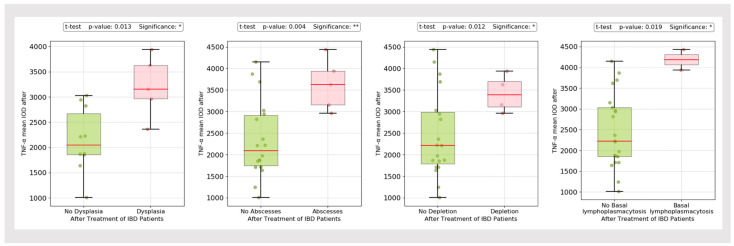
TNF-α mean IOD in IBD patients (dysplasia, abscesses, mucin depletion and basal plasmacytosis). * *p* < 0.05, ** *p* < 0.01.

The highest mean IOD TNF-α staining was observed in samples with dysplasia, abscesses, mucin depletion and basal plasmacytosis (Figure 2 and Figure 3).

Our group assessed the TNF-α mean IOD before and after treatment in responder versus non-responder patients. Responders were defined as those with a decrease of ≥3 points in the Mayo score (UC patients) and a decrease of 2 points in the Harvey Bradshaw Index (HBI) score from baseline values (CD patients), according to definitions previously used in other clinical trials. [50,51].

**Figure 4:**
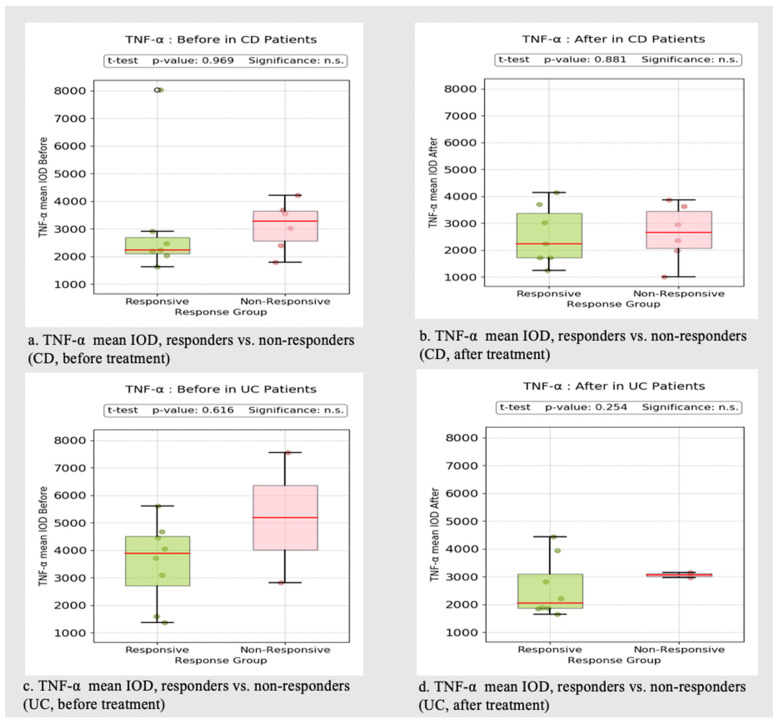
TNF-α mean IOD before and after treatment. °: outliers, n.s.: no significance.

Relatively surprisingly, the comparison of TNF-α staining between the two groups did not lead to statistically significant differences, despite non-responders exhibiting a slightly higher TNF-α mean IOD before treatment in UC patients. In CD patients, the difference in IHC staining was almost negligible. The post-treatment values were relatively similar in CD (Figure 4).

**Figure 5:**
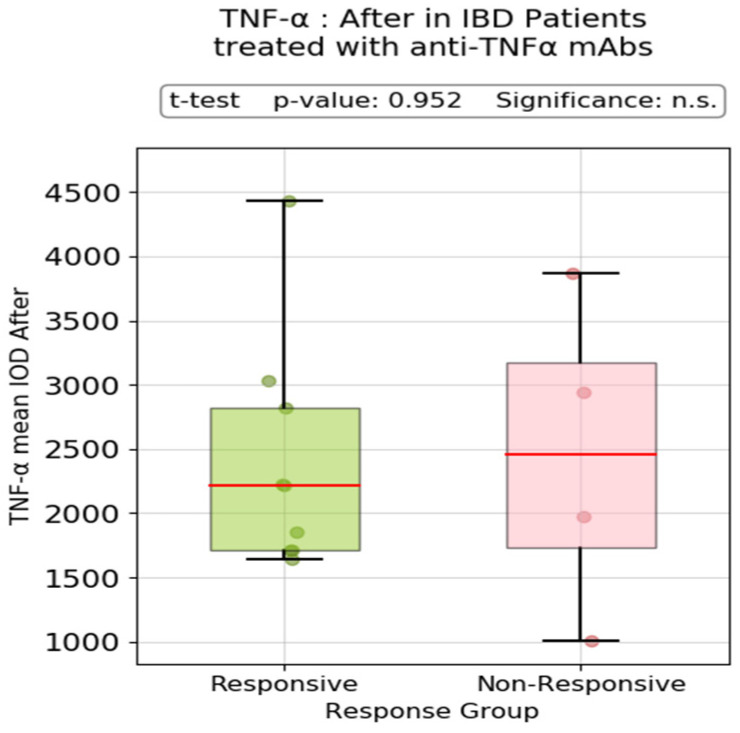
Post-treatment TNF-α mean IOD in patients treated with anti-TNF-α therapy (responders vs. non-responders). n.s.: no significance.

Neither a similar comparison in the subgroup of patients treated with anti-TNF-α therapy (Infliximab and Adalimumab) nor significant post-treatment variation was observed (Figure 5).

**Figure 6:**
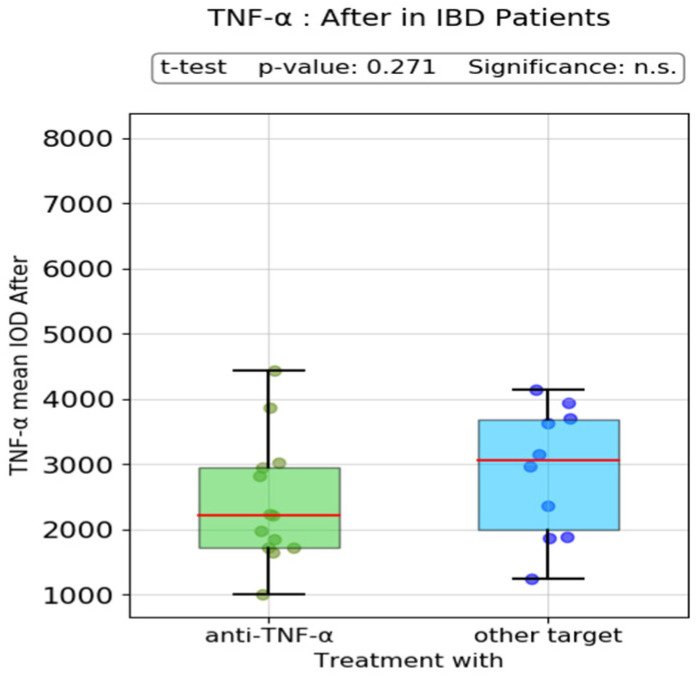
Post-treatment TNF-α mean IOD in patients treated with other advanced therapies. n.s.: no significance.

The scatter plot analysis of post-treatment TNF-α mean IOD revealed no significant difference between patients treated with anti-TNF-α therapy versus other novel advanced therapies (i.e., anti-interleukin (IL)-12/23, anti-α_4_β_7_ integrin antibody or JAK inhibitor molecules) (Figure 6).

**Figure 7:**
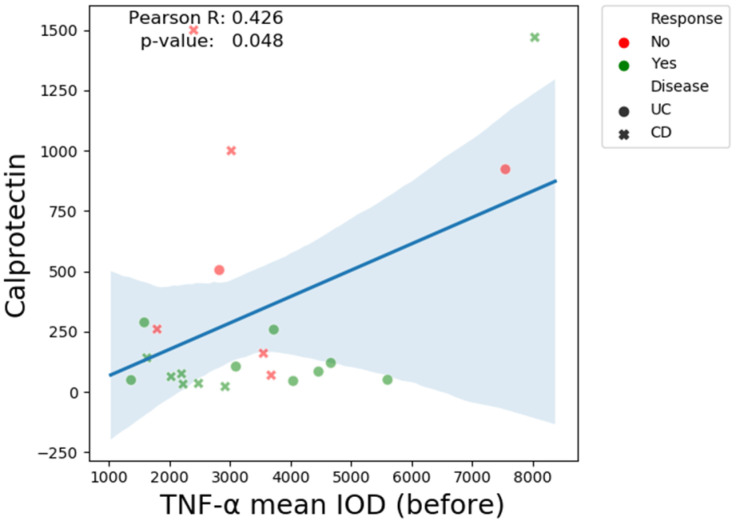
The Pearson correlation coefficient TNF-α mean IOD (before treatment) and fecal calprotectin.

**Figure 8:**
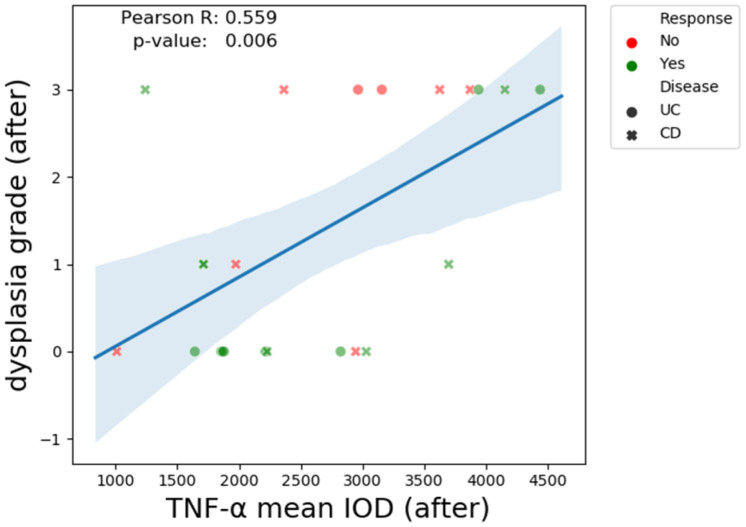
The Pearson correlation coefficient TNF-α mean IOD (after treatment) and dysplasia grade.

There is a clear positive correlation (R = 0.42, *p*-value = 0.04) between fecal calprotectin and pre-treatment TNF-α mean IOD (Figure 7). A similar relationship is observed between post-treatment TNF-α and dysplasia grade (R = 0.55, *p*-value = 0.006) (Figure 8).

#### 3.2.2. Interleukin-1β and Interleukin-6

**Figure 9:**
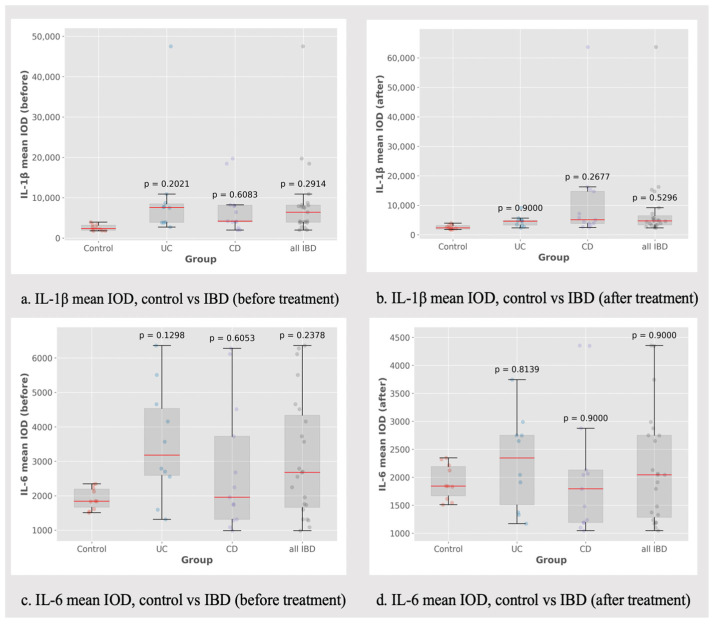
IL-1β and IL-6 mean IOD (control group vs. IBD group).

The mucosal expression of IL-1β and IL-6 was relatively steady between the initial and post-treatment evaluation, without major discrepancies (Figure 9).

**Figure 10:**
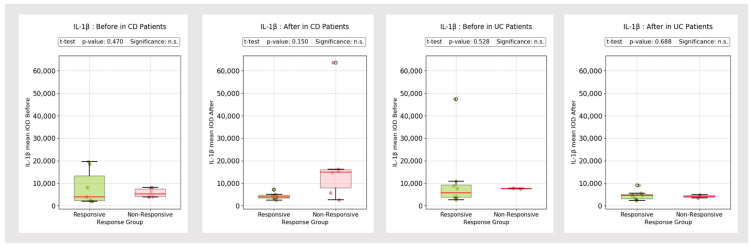
IL-1β mean IOD (control group vs. IBD group). °: outliers, n.s.: no significance.

**Figure 11:**
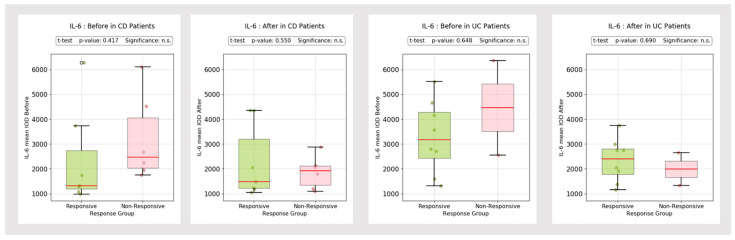
IL-6 mean IOD (control group vs. IBD group). °: outliers, n.s.: no significance.

The mean IL-1β post-treatment IOD values tended to be slightly higher in non-responder CD patients compared to responders, but there was only a subtle difference (Figure 10). At the same time, the IL-6 IOD values remained relatively steady between pre- and post-treatment evaluations in both UC and CD patients (Figure 11).

**Figure 12:**
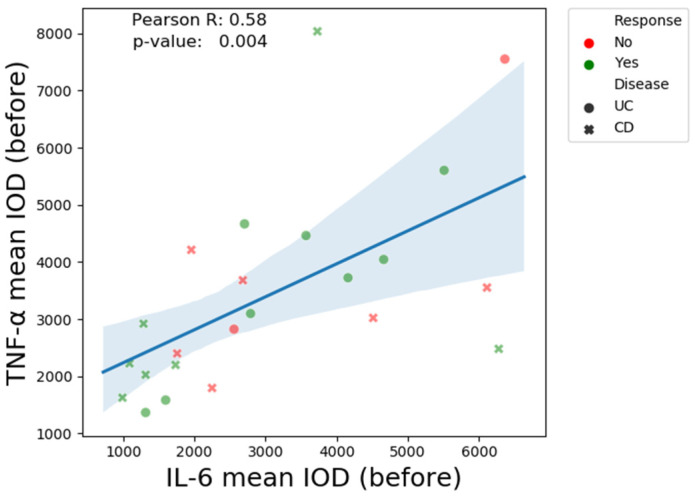
The Pearson correlation coefficient mean IOD values of IL-6 and TNF-α.

We observed a positive correlation (R = 0.58, *p*-value = 0.004) between the immunohistochemical mean IOD values of IL-6 and TNF-α (Figure 12).

#### 3.2.3. Interleukin-10

**Figure 13:**
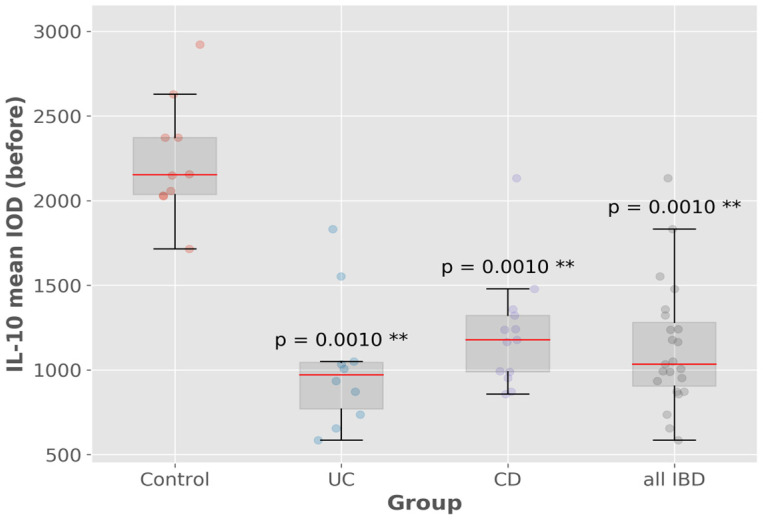
IL-10 mean IOD (control group vs. IBD group). ** *p* < 0.01.

The IL-10 mean IOD values were remarkably higher in the control group compared to both UC and CD patients (*p*-value = 0.001) (Figure 13).

**Figure 14:**
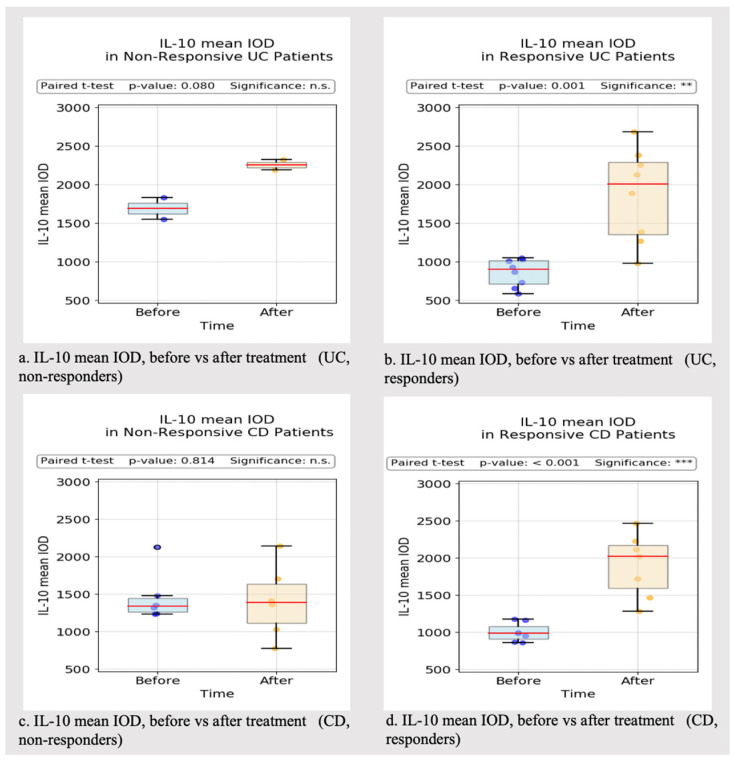
IL-10 mean IOD values (responder vs. non-responder IBD patients). ** *p* < 0.01, *** *p* < 0.001, n.s.: no significance.

In the responder group, the post-treatment IL-10 mean IOD was significantly higher compared to the index evaluation in both CD and UC patients (*p*-value = 0.001). In non-responders, the IOD values were relatively similar in CD patients before and after treatment, whereas the IOD values were slightly higher in UC patients, despite not reaching statistical significance (*p*-value = 0.08) (Figure 14).

**Figure 15:**
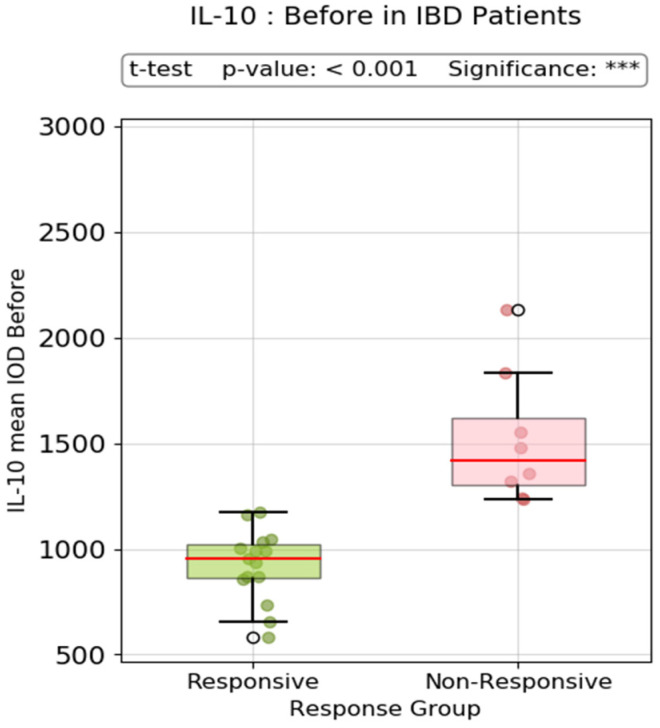
Pre-treatment IL-10 mean IOD values (responder vs. non-responder IBD patients). °: outliers, *** *p* < 0.001.

The pre-treatment IL-10 mean IOD values were significantly lower in responder versus non-responder IBD patients, with a *p*-value < 0.001 (Figure 15).

**Figure 16:**
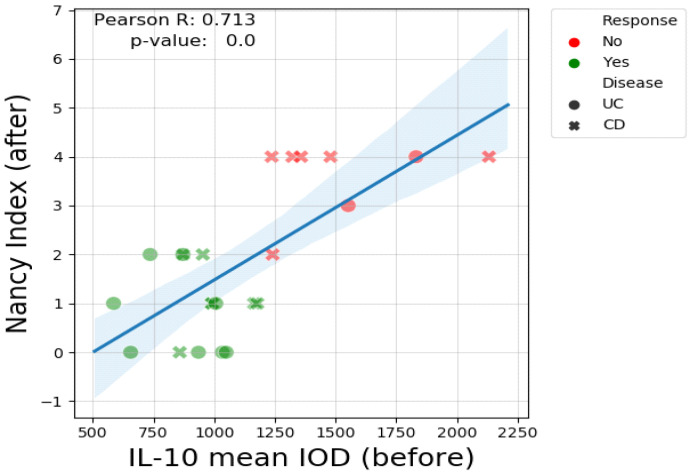
The Pearson correlation coefficient, pre-treatment IL-10 and post-treatment Nancy Index.

Notably, pre-treatment IL-10 levels positively correlate with the Nancy Index after treatment (*p*-value < 0.001), and may therefore predict failure of biologic therapy (Figure 16).

**Figure 17:**
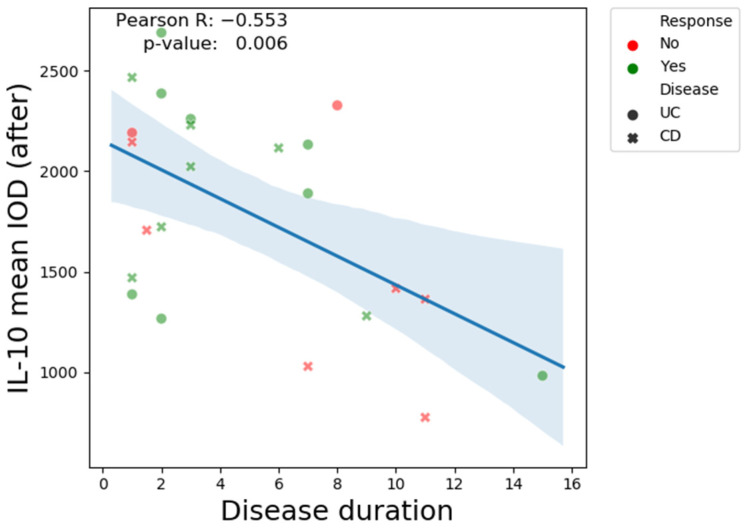
The Pearson correlation coefficient, post-treatment IL-10 and disease duration.

At the same time, there was a negative correlation between post-treatment IL-10 levels and disease duration. High IOD values seem to be associated with a longer disease duration (Figure 17).

#### 3.2.4. Immunohistochemistry of Dysplastic Markers

**Figure 18:**
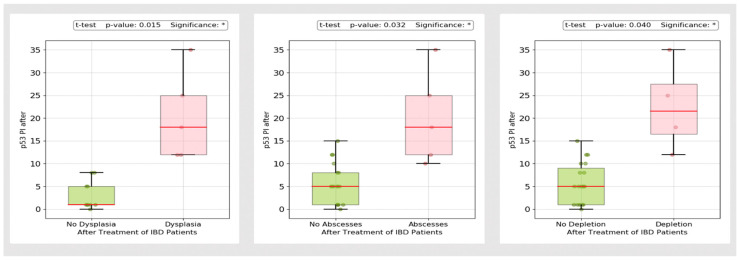
p53 immunostaining in patients with dysplasia, abscesses and mucin depletion. * *p* < 0.05, n.s.: no significance.

**Figure 19:**
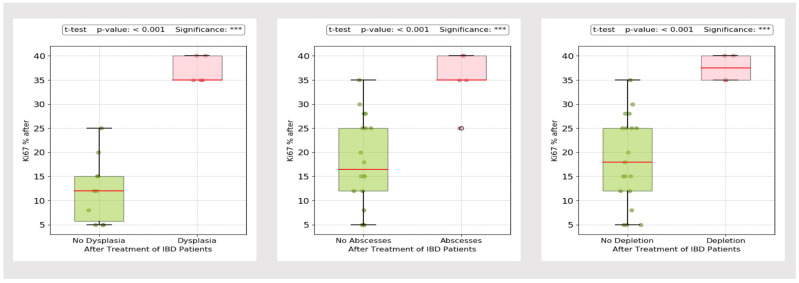
Ki-67 immunostaining in patients with dysplasia, abscesses and mucin depletion. °: outliers, *** *p* < 0.001.

Chronic inflammation is a risk factor for the development of varying degrees of dysplasia. We assessed Ki-67 and p53. As shown in Figure 18 and Figure 19, histological inflammatory burden appears to have a strong influence on the staining pattern of both Ki-67 and p53, with overexpression detected in patients with abscesses (*p*-value = 0.03 and <0.001 for p53 and Ki-67, respectively), mucin depletion (*p*-value = 0.04 and <0.001 for p53 and Ki-67, respectively) and dysplasia (*p*-value < 0.001).

**Figure 20:**
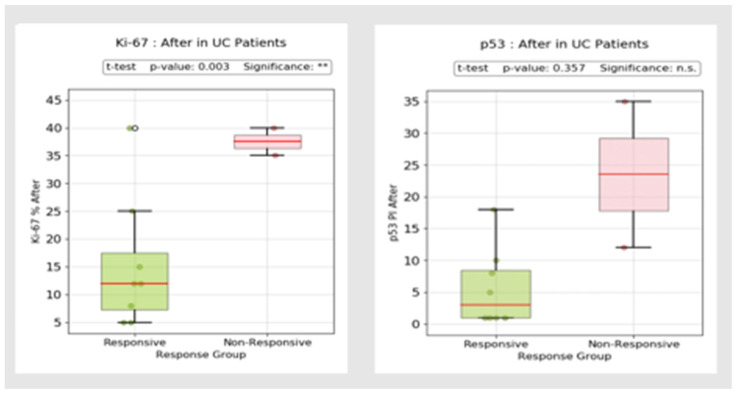
p53 and Ki-67 post-treatment immunostaining (responders vs. non-responders in UC patients). °: outliers, ** *p* < 0.01, n.s.: no significance.

When comparing post-treatment immunostaining patterns in UC patients, both p53 and Ki-67 exhibited lower expression in the responder group, although statistical significance was observed only for the Ki-67 proliferation index (Figure 20).

## 4. Discussion

Due to recent advances in molecular biology and the recognition of multiple immunologic pathways in IBD, novel treatments have emerged in the last decade, with many others being scrutinized in clinical trials [52,53,54,55,56]. Unfortunately, most therapies demonstrate a response rate of under 60%, which tends to diminish after each drug failure, particularly if primary failure is the reason for withdrawal [57,58].

The long-term goals have become more stringent, and histologic healing seems to ensure a better prognosis compared to mucosal healing alone [59,60,61]. Persistent microscopic inflammation is associated with increased relapse rates, hospitalization and colectomy [62,63]. The background of this inflammatory state is a milieu of mucosal cytokines, with well-characterized concerted action in both UC [64,65] and CD [66,67]. Distinct mucosal profiles of cytokines are produced in different phases of IBD, whether quiescent or active disease [68].

TNF- α is a central cytokine in IBD pathophysiology and anti-TNF-α therapy reduces inflammation and aids mucosal healing [69]. In our study, UC patients had higher TNF-α expression compared to CD patients. There is no clear data comparing the level of mucosal TNF-α in UC versus CD patients, but prior analysis has suggested that TNF-α levels in the inflamed mucosa before treatment could predict treatment response, whereas post-treatment levels could be considered an index of the efficacy of anti-TNF-α treatment [30,70,71]. TNF-α mucosal levels seem to have superior accuracy as a biomarker for disease activity compared to serum TNF-α [72], and are considered a promising tool in the era of precision medicine [73,74].

In UC patients, mucosal TNF-α correlates with UCDAI (UC disease activity index) [75], whereas a recent study suggested that mucosal TNF-α could predict the need for biologic therapy within the first year after UC diagnosis [76]. Moreover, pre-treatment mucosal TNF-α might have a predictive role in assessing remission and mucosal healing rates in anti-TNF-α therapy, whereas patients with intense TNF-α expression need a higher dose of Infliximab therapy [77]. Conversely, a normal post-treatment level is associated with a long relapse-free period after biologic therapy withdrawal [73]. Similar observations are available for CD patients [78]. Noticeably, the prognostic role of TNF-α levels is not strictly limited to anti-TNF-α therapy. Murate et al. reported that high serum-TNF-α can predict the clinical response to ustekinumab [79].

In our study, TNF-α expression was significantly higher in UC compared to CD patients. The highest IOD was observed in patients with dysplasia, abscesses, mucin depletion and basal plasmacytosis. Non-responders had higher pre- and post-treatment TNF-α expression in both UC and CD compared to responders. On the contrary, the same analysis conducted in the subpopulation treated with anti-TNF-α therapy (Infliximab and Adalimumab) did not reveal a substantial difference in TNF-α expression between responders and non-responders. We also divided our cohort in two subgroups according to the treatment-approach: IBD patients undergoing anti-TNF-α therapy versus other novel therapies (anti-integrin agents, including Vedolizumab, anti-interleukin agents, including Ustekinumab (a monoclonal antibody directed to IL12/23p40) and anti-JAK inhibitors, including Tofacitinib). The post-treatment TNF-α IOD between the two subgroups was relatively similar, according to the ANOVA analysis.

IL-1β and IL-6 are important cytokines for both intestinal inflammation and colorectal cancer [80]. IL-1β is an important mediator of inflammation and tissue damage in IBD, as it contributes to the recruitment and activation of immune cells to the mucosa [81]. A positive association between mucosal inflammation severity and IL-1β levels has been reported [82]. Anakinra inhibits the activity of IL-1β, but the results from other autoimmune diseases have not been extrapolated to IBD [83]. One plausible explanation resides in the fact that IL-1β acts in conjunction with other major pro-inflammatory cytokines (IL-6 and TNF-α) to induce IBD inflammation and it is not the dominant driver of inflammation [84]. IL-6 has pleiotropic effects on the immune system [85], with dual and contrasting effects depending on the stage of disease. IL-6 acts during initiation of the inflammatory process to induce the recruitment of PMNs and macrophages for wound healing processes. Conversely, IL-6 also promotes cytokine secretion by Th1, Th2 or Th17 cells [86]. Blockade of IL-6 resulted in a significant clinical response and induction of remission in moderate-to-severe CD patients with previous failure of anti-TNF-α therapy [87], albeit at the cost of increased adverse events related to excessive immunosuppression [56].

In our cohort, we observed a positive correlation between IL-6 and TNF-α mean IOD values. The assessment of IL-1β quantified similar pre- and post-treatment values in UC, whereas post-treatment CD values were higher in non-responders.

Upon examining IL-10 immunostaining, we observed the most compelling results. IL-10 exerts immunosuppressive effects on dendritic cells and macrophages by inhibiting their role to stimulate effector T cells [88,89]. Genetic variations of IL-10 are associated with the early onset of IBD [90,91,92], hinting at the importance of IL-10 in controlling immunological gut homeostasis [93]. Although the augmentation of IL-10 activity was considered a potential treatment target, no major effect from administering IL-10 has been detected [94]. It has been reported that anti-TNF-α mAb therapy increases IL-10 production in macrophages, and the efficacy of anti-TNF-α therapy depends on IL-10 signaling in macrophages, rather than T cells [95]. Therefore, a reduced response to IL-10 might explain the primary non-response to anti-TNF-α therapy [95].

The control group had markedly elevated levels of mucosal IL-10 expression compared to the IBD patients. In the IBD group, non-responder patients had higher pre-treatment levels compared to responders. The post-treatment IL-10 expression in this category was relatively similar in CD and only slightly elevated in UC, although this was without statistical significance (p-value = 0.08). Conversely, in the responder group, both UC and CD patients had very low pre-treatment IL-10 immunopositivity and a noticeable elevation in post-treatment IOD values (*p*-value = 0.001). Although it might not appear very intuitive, high pre-treatment levels of IL-10, which is regarded as one of the most important anti-inflammatory cytokines, are a negative predictive factor for response. On the other hand, patients with moderate-to-severe clinical and endoscopic disease, but with low expression of IL-10, seem to have a better prognosis due to a favorable treatment response. There was a statistically significant difference (*p*-value < 0.001) in pre-treatment IL-10 mean IOD values between responders and non-responders in IBD patients (Figure 15). A very interesting observation can be made when assessing the relationship between pre-treatment IL-10 IOD values and post-treatment Nancy Index. There was a clear positive correlation (Pearson R value = 0.71) between these two indices, supporting the idea that high IL-10 immunohistochemical staining before treatment predicts failure of biologic therapy. The reverse scenario is depicted in the scatter plot showing the negative correlation between post-treatment IL-10 values and disease duration. Practically, a prolonged disease course is associated with weak IL-10 expression in post-treatment biopsy samples, which, at least in our study, is a feature of non-responsiveness. As previously shown in other studies, the benefit of biologic therapy during the induction phase is significantly greater among patients with a short disease duration (mostly < 18 months) [96,97,98]. From previous studies we already know that a long disease course is a negative predictive factor in both CD [99] and UC [100], but the intricate mechanism has not been fully elucidated. IL-10 might be one of the explanations.

Since colorectal cancer (CCR) is one of the most feared complications of IBD, we also assessed the p53 positivity index and Ki-67 proliferation index. Despite a decreasing incidence, the estimated risk of CRC remains approximately two-fold higher compared to the general population [101]. Disease extent, severity and inflammatory burden are major contributing factors [102]. Expression of p53 serves as a reliable biomarker in the early stages of UC-associated colon carcinogenesis [103]. Overexpression can be detected even in the absence of dysplasia and is a useful tool for pathologists to differentiate between regenerative changes and intraepithelial neoplasia [103]. In our study, overexpression was observed in patients with dysplasia, abscesses and mucin depletion, with a substantial difference in immunostaining patterns between responders and non-responders. In our opinion, patients with significant p53 and Ki-67 IHC reactivity should undergo strict surveillance, even in the absence of other high-risk features (such as extensive colitis, stricture, primary sclerosing cholangitis or family history of CRC). Previous studies have shown progression to CRC in patients with indefinite dysplasia and p53 overexpression [104].

We acknowledge that the small sample size is an important limitation of our study, but hopefully it will trigger interest for future studies directed towards research in the area of tailoring medical management in inflammatory bowel diseases.

## 5. Conclusions

The medical management of inflammatory bowel diseases has changed dramatically in recent years, with many molecules already approved and a multitude of others under scrutiny in clinical trials. However, there is a significant gap in the literature regarding a more tailored approach, which is the essence of precision medicine. Our study delved into the immunohistochemical analysis of inflammatory and dysplastic markers, aiming to prospectively evaluate the impact of biologic therapy during treatment. High pre-treatment interleukin-10 expression is associated with failure of biologic therapy, histological inflammatory activity and longer disease duration. It serves as a useful tool to identify a high-risk subset of IBD patients and guide a more intensive monitoring strategy.

## Figures and Tables

**Table 1:** IHC analysis—antibodies, clones, dilutions, antigen retrievals and external positive controls.

Antibody	Clones	Dilution	Antigen Retrieval	External Positive Control
IL-1β	Polyclonal	1:50	citrate buffer, pH 6	Spleen
IL-6	Monoclonal 8H12	1:50	citrate buffer, pH 6	Spleen
TNF-α	Polyclonal	1:300	citrate buffer, pH 6	Spleen
IL-10	Polyclonal	1:100	Tris-EDTA buffer, (HIER), pH 8	Prostate adenocarcinoma
Ki-67	Monoclonal MIB-1	1:75	citrate buffer, pH 6	Palatine tonsils
p53	Monoclonal DO-7	1:75	Tris-EDTA buffer (HIER), pH 9	Palatine tonsils

**Table 2:** Study population data.

	UC	CD	All IBD
Number	20	26	46
Disease Duration (median, 5–95%)	2.5 (1.0–11.8)	3.0 (1.0–11.0)	3.0 (1.0–11.0)
Sex			
M	60.0%	46.2%	52.2%
F	40.0%	53.8%	47.8%
Current Biological Treatment			
Vedolizumab	20.0%	38.5%	30.4%
Infliximab	40.0%	38.5%	39.1%
Adalimumab	10.0%	23.1%	17.4%
Tofacitinib	20.0%	none	8.7%
Ustekinumab	10.0%	none	4.3%

**Table 3:** IBD phenotype according to the Montreal classification.

Ulcerative Colitis Phenotype			
E1		10.0%	
E2		40.0%	
E3		50.0%	
Crohn’s Disease Phenotype			
L1B1	7.7%		30.8%
L1B2	15.4%		
L1B3	7.7%		
L2B2	30.8%		38.5%
L2B2	7.7%		
L3B1	7.7%		30.8%
L3B2	15.4%		
L3B3	7.7%		

## Data Availability

Data that support the findings of this study and materials are available from the first author upon request.

## Abbreviations

The following abbreviations are used in this manuscript:

IBD	Inflammatory bowel disease
CD	Crohn’s disease
UC	Ulcerative colitis
IHC	Immunohistochemistry
IOD	Integrated optical density
TNF	Tumor necrosis factor
mAB	Monoclonal antibody
IL	Interleukin
